# Association of Visceral Fat Area, Smoking, and Alcohol Consumption with Reflux Esophagitis and Barrett's Esophagus in Japan

**DOI:** 10.1371/journal.pone.0133865

**Published:** 2015-07-30

**Authors:** Juntaro Matsuzaki, Hidekazu Suzuki, Masao Kobayakawa, John M. Inadomi, Michiyo Takayama, Kanako Makino, Yasushi Iwao, Yoshinori Sugino, Takanori Kanai

**Affiliations:** 1 Center for Preventive Medicine, Keio University Hospital, Tokyo, Japan; 2 Division of Gastroenterology and Hepatology, Department of Internal Medicine, Keio University School of Medicine, Tokyo, Japan; 3 Department of Gastroenterology, National Center for Global Health and Medicine, Tokyo, Japan; 4 Division of Gastroenterology, Department of Internal Medicine, University of Washington Medical School, Seattle, Washington, United States of America; Graduate School of Medicine, Osaka University, JAPAN

## Abstract

**Background:**

Central obesity has been suggested as a risk factor for gastroesophageal reflux disease. The aim of this study was to evaluate the association of visceral fat area and other lifestyle factors with reflux esophagitis or Barrett’s esophagus in Japanese population.

**Methods:**

Individuals who received thorough medical examinations including the measurement of visceral fat area by abdominal computed tomography were enrolled. Factors associated with the presence of reflux esophagitis, the severity of reflux esophagitis, or the presence of Barrett’s esophagus were determined using multivariable logistic regression models.

**Results:**

A total of 2608 individuals were eligible for the analyses. Visceral fat area was associated with the presence of reflux esophagitis both in men (odds ratio, 1.21 per 50 cm^2^; 95% confident interval, 1.01 to 1.46) and women (odds ratio, 2.31 per 50 cm^2^; 95% confident interval, 1.57 to 3.40). Current smoking and serum levels of triglyceride were also associated with the presence of reflux esophagitis in men. However, significant association between visceral fat area and the severity of reflux esophagitis or the presence of Barrett’s esophagus was not shown. In men, excessive alcohol consumption on a drinking day, but not the frequency of alcohol drinking, was associated with both the severity of reflux esophagitis (odds ratio, 2.13; 95% confident interval, 1.03 to 4.41) and the presence of Barrett’s esophagus (odds ratio, 1.71; 95% confident interval, 1.14 to 2.56).

**Conclusion:**

Visceral fat area was independently associated with the presence of reflux esophagitis, but not with the presence of Barrett’s esophagus. On the other hand, quantity of alcohol consumption could play a role in the development of severe reflux esophagitis and Barrett’s esophagus in Japanese population.

## Introduction

Esophageal adenocarcinoma is an aggressive malignancy with a poor prognosis. In the Western countries, the incidence of esophageal adenocarcinoma has increased dramatically in the last three decades.[1] Although there also appears to be a trend toward an increase in the incidence of adenocarcinoma in Asia, the absolute incidence remains relatively low.[2] Even in the Unites States, increasing trends in incidence rates of esophageal adenocarcinoma differed by race/ethnicity: 1.8% per year in white men, 2.1% per year in white women, 2.8% per year in Hispanic men, while no significant changes in other racial/ethnic groups.[3] This suggests that risk stratification for the progression to esophageal adenocarcinoma should be independently assessed for each race.

Barrett’s esophagus (BE) is the presumed precursor lesion of esophageal adenocarcinoma. Previously diagnosed reflux esophagitis (RE) also increases the risk of esophageal adenocarcinoma.[4] In the Western countries, it has been generally accepted that central obesity is associated with RE and BE.[5–8] However, the evidences supporting the association of central obesity with RE or BE are limited in Asia.[9] The aim of the present study was to assess the association of central obesity by the measurement of visceral fat area at the level of the navel with RE or BE. Other lifestyle-related risk factors were also assessed and adjusted using multivariable analyses. Since the incidence of BE and esophageal adenocarcinoma was highly different between genders,[10] analyses were conducted for each gender separately.

## Materials and Methods

### Ethics statement

Since the present study was non-intrusive retrospective research, informed consent for each participant was not obtained. The records of participants were anonymized and de-identified prior to analysis. The protocol for this study was approved by the ethics committee of the Keio University School of Medicine (No. 20130501).

### Study population and settings

Individuals who received esophagogastroduodenoscopy (EGD) and thorough medical examinations at Keio University Hospital between October 2012 and November 2013 were enrolled. Thorough medical examinations included questionnaires of medical history and lifestyle habit, physical examination, complete blood count, serum chemistries, computed tomography (CT) scans of the chest and the abdomen, abdominal ultrasound, and EGD. Individuals who had undergone esophageal or gastric resection before the medical examinations were excluded from the study. Those with incomplete questionnaires were also excluded.

### Definitions of endoscopic findings

RE was diagnosed and graded according to the Los Angeles classification.[11] BE was defined as an esophagus in which any portion of the normal distal squamous epithelial lining was replaced by columnar epithelium that was clearly visible endoscopically (≥1cm) above the gastroesophageal junction according to the British Society of Gastroenterology guidelines on the diagnosis and management of BE.[12] Irregular Z-line which shows a tongue of columnar-lined esophagus shorter than 1 cm was not diagnosed as BE.[12] Hiatus hernia was defined as a circular extension of the gastric mucosa above the diaphragmatic hiatus.

### Data collection

We obtained information on age, sex, body mass index (BMI, kg/m^2^), waist circumference at the level of the navel during minimal respiration, cigarette smoking status, alcohol consumption, medication, and sleeping hours from the questionnaires. In terms of alcohol consumption, frequency of alcohol drinking (number of days per week) and quantity consumed on days during which drinking occurred (gram ethanol per drinking day) were asked separately. The frequency and intensity of daily exercise, such as sweating for more than 30 minutes twice a week or walking for one hour or more in a day, and eating habits, such as skipping breakfast 3 times a week, having late evening snack 3 times a week, and having late dinner 3 times a week, were also asked using questionnaires. Blood pressure and blood markers including triglyceride (TG), high-density lipoprotein cholesterol (HDL-C), low-density lipoprotein cholesterol (LDL-C), hemoglobin A1c (Hb-A1c), homeostatic model assessment of insulin resistance (HOMA-R), and uric acid (UA) were measured. In addition, visceral fat area (VFA) was measured at the navel level using a single-slice CT scan (Aquilion CXL; Toshiba Medical Systems Corporation, Tochigi, Japan).

### Statistical analysis

Analyses were conducted for each gender separately. Factors associated with the presence of RE, the severity of RE (Los Angeles grade A vs. B to D), or the presence of BE were evaluated. For univariable analyses, continuous variables were compared using Student's t-test. The chi-square test or Fisher's exact test was used to compare categorical variables. Subsequently, factors that showed marginal differences (p < 0.2) in univariable analyses were included in age-adjusted logistic regression models. The comparison unit for a continuous variable was set to be close to the standard deviation of each factor. Finally, marginally associated factors (p < 0.1) in age-adjusted logistic regression analyses were included in multivariable logistic regression models with forward stepwise variable selection. Subgroup analyses were also conducted based on VFA (< 100 cm^2^ or ≥ 100 cm^2^) in the same way.

The continuous variables measured in this study were expressed as mean ± standard deviation. All tests for statistical significance were two-sided at p = 0.05. All statistical analyses were conducted using IBM SPSS Statistics version 22 (IBM Japan, Tokyo, Japan).

## Results

### Participant characteristics

**Fig 1:**
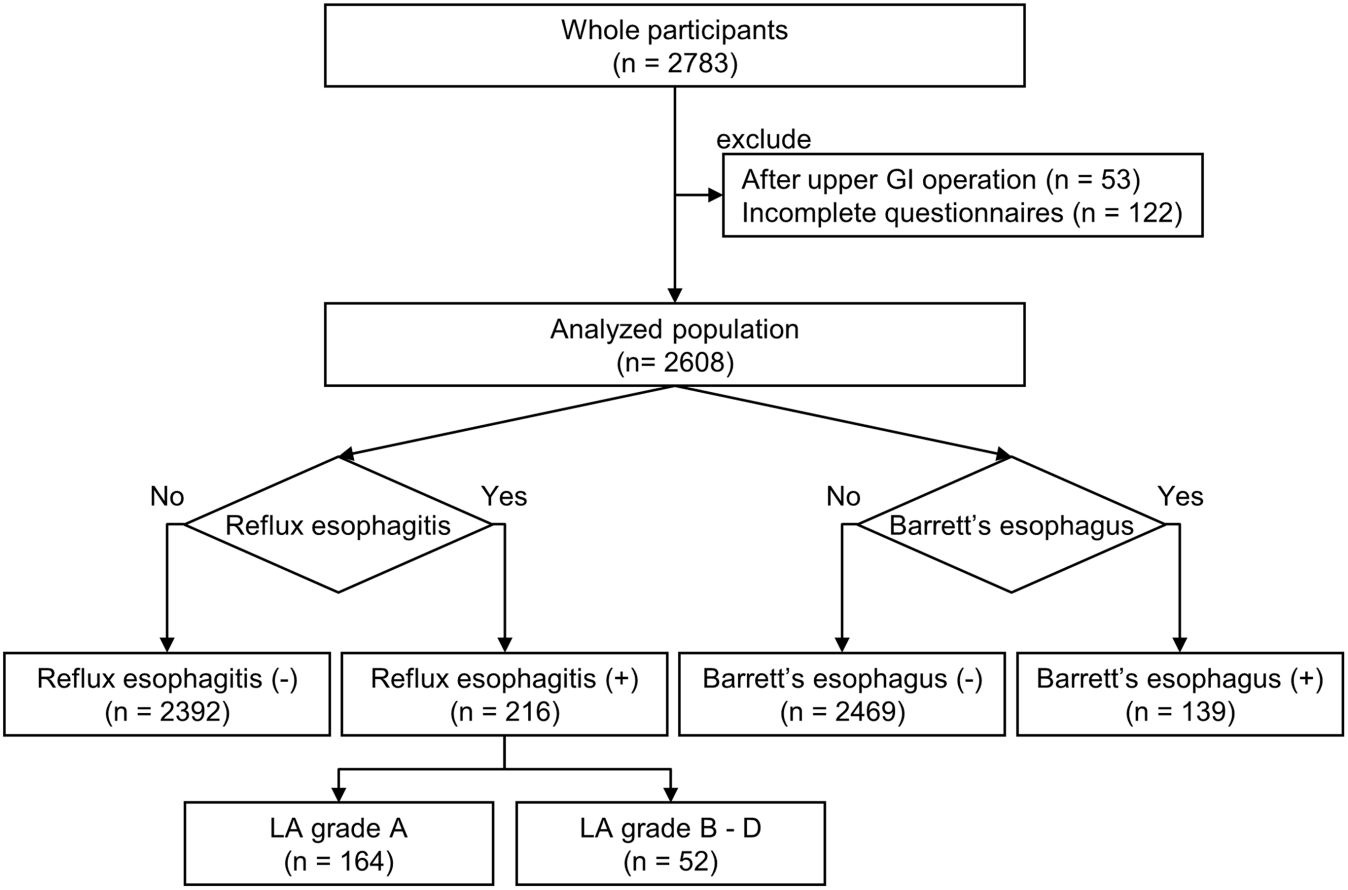
Diagram of inclusion of the study population.

A total of 2,783 individuals underwent thorough medical examinations including EGD during the study period (Fig 1). After we excluded 175 individuals who did not meet the inclusion criteria, 2,608 individuals (1,625 men and 983 women) were evaluated in this analysis. RE and BE were diagnosed in 216 (8.3%) and 139 (5.3%) individuals, respectively. Those with RE were divided into 164 individuals with mild RE and 52 with severe RE. In our study, all of BE were short-segment BE (SSBE). The raw data can be found in Supporting Information (S1 Raw Data).

### Factors associated with the presence of reflux esophagitis

Differences between individuals with and without RE were investigated using the univariable analyses (Table 1). Individuals with RE were younger than those without RE in men (p = 0.007), but not in women (p = 0.06). Both in men and women, obesity-related markers such as BMI, waist circumference, and VFA were associated with the presence of RE. Individuals whose body weight had increased more than 10 kg compared with their weight at 20 years of age were more likely to have RE. Serum TG levels were higher, and HDL-C levels were lower in those with RE than those without RE. In women, serum LDL-C levels and Hb-A1c levels were also higher in those with RE. HOMA-R levels were higher in those with RE in both men and women. In men, serum uric acid levels were higher in those with RE.

**Table 1:** Differences between participants with and without reflux esophagitis.

		Men			Women		
		RE (-) (n = 1443)	RE (+) (n = 182)	p value	RE (-) (n = 949)	RE (+) (n = 34)	p value
Age (y)		60.8 ± 12.9	58.0 ± 12.7	**0.007** ‡	58.7 ± 13.4	63.0 ± 10.4	0.06‡
BMI (kg/m^2^)		24.0 ± 3.0	24.7 ± 2.8	**0.001** ‡	21.6 ± 3.3	23.9 ± 4.1	**0.002** ‡
Waist circumference (cm)		83.3 ± 8.4	85.7 ± 7.7	**<0.001** ‡	78.3 ± 10.0	85.9 ± 11.1	**<0.001** ‡
Visceral fat area (cm^2^)		103 ± 47	117 ± 47	**<0.001** ‡	64 ± 38	105 ± 46	**<0.001** ‡
≥ 10kg of BW increase since 20 y/o		729 (51%)	117 (64%)	**0.001** ¶	251 (26%)	16 (47%)	**0.01** ¶
Systolic BP (mmHg)		121 ± 16	122 ± 15	0.43‡	115 ± 19	123 ± 16	**0.02** ‡
Diastolic BP (mmHg)		77 ± 10	79 ± 11	**0.02** ‡	73 ± 10	77 ± 10	0.08‡
TG (mg/dL)		119 ± 74	144 ± 115	**0.004** ‡	90 ± 64	125 ± 86	**0.002** ‡
HDL-C (mg/dL)		52.8 ± 12.8	50.5 ± 13.4	**0.03** ‡	64.4 ± 14.6	58.9 ± 13.4	**0.03** ‡
LDL-C (mg/dL)		114 ± 28	117 ± 30	0.18‡	118 ± 30	130 ± 37	**0.02** ‡
Hb-A1c (%)		5.7 ± 0.7	5.7 ± 0.6	0.52‡	5.6 ± 0.4	5.8 ± 0.6	**0.02** ‡
HOMA-R		1.7 ± 1.4	2.0 ± 1.4	**0.002** ‡	1.3 ± 1.0	1.8 ± 1.3	**0.03** ‡
UA (mg/dL)		6.2 ± 1.2	6.4 ± 1.3	**0.049** ‡	4.8 ± 1.1	4.9 ± 0.9	0.37‡
Smoking habit	Non-smoker	461 (32%)	47 (26%)	**0.001** †	759 (80%)	27 (79%)	0.88†
	Ex-smoker	764 (53%)	88 (48%)		138 (15%)	5 (15%)	
	Current smoking < 20 /day	93 (6%)	17 (9%)		39 (4%)	1 (3%)	
	Current smoking 20–39 /day	112 (8%)	24 (13%)		13 (1%)	1 (3%)	
	Current smoking ≥ 40 /day	13 (1%)	6 (3%)		0 (0%)	0 (0%)	
Quantity of alcohol consumption	< 40g ethanol/day	1006 (70%)	114 (63%)	0.06 ¶	887 (94%)	31 (91%)	0.49 ¶
	≥ 40g ethanol/day	437 (30%)	68 (37%)		62 (7%)	3 (9%)	
Frequency of alcohol drinking	None or social	409 (28%)	46 (25%)	0.28†	599 (63%)	24 (71%)	0.63†
	1–4 days /week	410 (28%)	46 (25%)		199 (21%)	5 (15%)	
	5–7 days / week	624 (43%)	90 (50%)		151 (16%)	5 (15%)	
Exercise	Sweating for 30 mins twice a week	607 (42%)	67 (37%)	0.20¶	314 (33%)	7 (21%)	0.14¶
	≥ 1 hour walk / day	507 (35%)	63 (35%)	0.93¶	307 (32%)	12 (35%)	0.71¶
Eating habit	No breakfast ≥ 3 times / week	265 (18%)	41 (23%)	0.19¶	137 (14%)	2 (6%)	0.21¶
	Late evening snack ≥ 3 times / week	171 (12%)	26 (14%)	0.34¶	146 (15%)	7 (21%)	0.47¶
	Late dinner ≥ 3 times / week	342 (24%)	57 (31%)	**0.03** ¶	151 (16%)	7 (21%)	0.48¶
Medication	Proton pump inhibitor	186 (13%)	26 (14%)	0.56¶	94 (10%)	4 (12%)	0.77¶
	Aspirin	142 (10%)	12 (7%)	0.18¶	34 (4%)	1 (3%)	1.00¶
	Statin	281 (20%)	29 (16%)	0.27¶	179 (19%)	10 (29%)	0.13¶
Sleeping < 6 hours/night		805 (56%)	119 (65%)	**0.01** ¶	559 (59%)	21 (62%)	0.86¶
Sleep apnea syndrome		54 (4%)	8 (4%)	0.68¶	5 (1%)	0 (0%)	1.00¶
Hiatus hernia		444 (31%)	145 (80%)	**<0.001** ¶	210 (22%)	27 (79%)	**<0.001** ¶
SSBE		82 (6%)	29 (16%)	**<0.001** ¶	23 (2%)	5 (15%)	**0.002**

Bold values indicate significant differences. RE, reflux esophagitis; BMI, body mass index; BW, body weight; BP, blood pressure; TG, triglyceride; HDL-C, high-density lipoprotein cholesterol; LDL-C, low-density lipoprotein cholesterol; Hb-A1c, hemoglobin A1c; HOMA-R, homeostatic model assessment of insulin resistance; UA, uric acid; SSBE, short-segment Barrett’s esophagus;

^¶^, Fisher’s exact test;

^†^, χ^2^test;

^‡^, Student’s t-test.

The rate of current smokers was higher in those with RE than in those without RE in men, but not in women. The rate of individuals who consumed more than 40 g ethanol on a day in which alcohol was consumed tended to be higher in those with RE than those without RE in men (p = 0.06), but not in women (p = 0.63). However, frequency of alcohol drinking was not associated with the presence of RE.

The rate of individuals who had a late dinner 3 times a week or more was higher in those with RE than without RE in men. The rate of individuals who slept less than 6 hours a night was also higher in those with RE in men. The presence of hiatus hernia or SSBE was associated with the presence of RE both in men and women.

### Factors associated with the severity of reflux esophagitis

Differences of characteristics between individuals with grade A esophagitis and those with grade B–D esophagitis were investigated using the univariable analyses (Table 2). In men, levels of VFA were slightly higher (p = 0.11) and levels of systolic blood pressure were higher (p = 0.04) in individuals with grade A esophagitis than those with grade B–D esophagitis. However, the other metabolic markers were not associated with the severity of RE. The rate of individuals who consumed more than 40 g ethanol on a drinking day tended to be higher in those with grade A esophagitis than those with grade B–D esophagitis in men (p = 0.11), but not in women (p = 1.00). The presence of hiatus hernia (p = 0.01) or SSBE (p = 0.01) was associated with the presence of RE in men. In women, all of individuals with grade B–D esophagitis had hiatus hernia, although the difference was not reached to the significance as compared with those with grade A esophagitis (p = 0.16).

**Table 2:** Differences between participants with mild and severe reflux esophagitis.

		Men			Women		
		RE, A (n = 138)	RE, B-D (n = 44)	p value	RE, A (n = 26)	RE, B-D (n = 8)	p value
Age (y)		57.7 ± 12.4	59.1 ± 13.5	0.53‡	61.5 ± 9.5	67.6 ± 12.6	0.15‡
BMI (kg/m^2^)		24.6 ± 2.6	25.3 ± 3.3	0.21‡	24.2 ± 4.3	23.0 ± 3.4	0.49‡
Waist circumference (cm)		85.2 ± 7.1	87.2 ± 9.5	0.22‡	87.3 ± 11.8	81.2 ± 7.1	0.18‡
Visceral fat area (cm^2^)		113 ± 43	129 ± 58	0.11‡	107 ± 48	97 ± 37	0.59‡
≥ 10kg of BW increase since 20 y/o		89 (65%)	28 (64%)	1.00¶	12 (46%)	4 (50%)	1.00¶
Systolic BP (mmHg)		120 ± 15	126 ± 16	**0.04** ‡	124 ± 16	120 ± 16	0.52‡
Diastolic BP (mmHg)		78 ± 11	81 ± 12	0.15‡	77 ± 10	76 ± 11	0.75‡
TG (mg/dL)		143 ± 126	147 ± 69	0.82‡	126 ± 95	118 ± 55	0.82‡
HDL-C (mg/dL)		50.6 ± 13.4	50.3 ± 13.6	0.90‡	59.0 ± 14.7	58.6 ± 8.5	0.94‡
LDL-C (mg/dL)		119 ± 30	111 ± 31	0.13‡	126 ± 22	141 ± 67	0.57‡
Hb-A1c (%)		5.7 ± 0.7	5.7 ± 0.5	1.00‡	5.8 ± 0.6	5.9 ± 0.3	0.68‡
HOMA-R		2.0 ± 1.4	2.2 ± 1.4	0.41‡	1.8 ± 1.3	1.9 ± 1.2	0.88‡
UA (mg/dL)		6.4 ± 1.3	6.6 ± 1.2	0.25‡	4.9 ± 1.0	5.2 ± 0.6	0.25‡
Smoking habit	Non-smoker	38 (28%)	9 (21%)	0.58†	20 (77%)	7 (88%)	0.87†
	Ex-smoker	66 (48%)	22 (50%)		4 (15%)	1 (13%)	
	Current smoking < 20 /day	13 (9%)	4 (9%)		1 (4%)	0 (0%)	
	Current smoking 20–39 /day	18 (13%)	6 (14%)		1 (4%)	0 (0%)	
	Current smoking ≥ 40 /day	3 (2%)	3 (7%)		0 (0%)	0 (0%)	
Quantity of alcohol consumption	< 40g ethanol/day	91 (66%)	23 (52%)	0.11¶	24 (92%)	7 (88%)	1.00¶
	≥ 40g ethanol/day	47 (34%)	21 (48%)		2 (8%)	1 (12%)	
Frequency of alcohol drinking	None or social	38 (28%)	8 (18%)	0.41†	18 (69%)	6 (75%)	0.31†
	1–4 days /week	35 (25%)	11 (25%)		5 (19%)	0 (0%)	
	5–7 days / week	65 (47%)	25 (57%)		3 (12%)	2 (25%)	
Exercise	Sweating for 30 mins twice a week	54 (39%)	13 (30%)	0.29¶	4 (15%)	3 (38%)	0.32¶
	≥ 1 hour walk / day	51 (37%)	12 (27%)	0.28¶	10 (39%)	2 (25%)	0.68¶
Eating habit	No breakfast ≥ 3 times / week	31 (23%)	10 (23%)	1.00¶	2 (8%)	0 (0%)	1.00¶
	Late evening snack ≥ 3 times / week	20 (15%)	6 (14%)	1.00¶	6 (23%)	1 (13%)	1.00¶
	Late dinner ≥ 3 times / week	40 (29%)	17 (39%)	0.26¶	5 (19%)	2 (25%)	1.00¶
Medication	Proton pump inhibitor	18 (13%)	8 (18%)	0.46¶	3 (12%)	1 (13%)	1.00¶
	Aspirin	10 (7%)	2 (5%)	0.73¶	1 (4%)	0 (0%)	1.00¶
	Statin	26 (19%)	3 (7%)	0.06¶	8 (31%)	2 (25%)	1.00¶
Sleeping < 6 hours/night		90 (65%)	29 (66%)	1.00¶	18 (69%)	3 (38%)	0.21¶
Sleep apnea syndrome		6 (4%)	2 (5%)	1.00¶	0 (0%)	0 (0%)	N. A.
Hiatus hernia		104 (75%)	41 (93%)	**0.01** ¶	19 (73%)	8 (100%)	0.16¶
SSBE		16 (12%)	13 (30%)	**0.01** ¶	3 (12%)	2 (25%)	0.57¶

Bold values indicate significant differences. RE, reflux esophagitis; BMI, body mass index; BW, body weight; BP, blood pressure; TG, triglyceride; HDL-C, high-density lipoprotein cholesterol; LDL-C, low-density lipoprotein cholesterol; Hb-A1c, hemoglobin A1c; HOMA-R, homeostatic model assessment of insulin resistance; UA, uric acid; SSBE, short-segment Barrett’s esophagus; N. A., could not be analyzed;

^¶^, Fisher’s exact test;

^†^, χ^2^ test;

^‡^, Student’s t-test.

### Factors associated with the presence of Barrett’s esophagus

Differences of characteristics between individuals with SSBE and those without SSBE were investigated using the univariable analyses (Table 3). In men, levels of VFA (p = 0.08), blood pressure (systolic, p = 0.09; diastolic, p = 0.03), LDL-C (p = 0.07), and HOMA-R (p = 0.06) were slightly higher in individuals with SSBE than those without SSBE. In women, the metabolic markers were not associated with the presence of SSBE. The rate of current smokers or ex-smokers tended to be higher in those with SSBE than in those without SSBE in men (p = 0.07), but not in women (p = 0.71). Both the quantity (p = 0.01) and frequency (p = 0.046) of alcohol consumption was associated with the presence of SSBE in men, but not in women. Proton pump inhibitor uses were greater in those with SSBE (p = 0.02) in men. The rate of individuals who slept less than 6 hours a night was also higher in those with SSBE in women (p = 0.03). The presence of hiatus hernia or RE was significantly associated with the presence of SSBE both in men and women.

**Table 3:** Differences between participants with and without short-segment Barrett’s esophagus.

		Men			Women		
		SSBE (-) (n = 1514)	SSBE (+) (n = 111)	p value	SSBE (-) (n = 955)	SSBE (+) (n = 28)	p value
Age (y)		60.4 ± 13.0	61.3 ± 10.9	0.42‡	58.7 ± 13.3	61.3 ± 12.4	0.31‡
BMI (kg/m^2^)		24.0 ± 3.0	24.5 ± 3.2	0.10‡	21.7 ± 3.4	21.2 ± 2.4	0.52‡
Waist circumference (cm)		83.5 ± 8.3	84.3 ± 9.0	0.33‡	78.6 ± 10.2	77.9 ± 6.5	0.60‡
Visceral fat area (cm^2^)		104 ± 47	112 ± 47	0.08‡	65 ± 39	69 ± 36	0.63‡
≥ 10kg of BW increase since 20 y/o		784 (52%)	62 (56%)	0.43¶	260 (27%)	7 (25%)	1.00¶
Systolic BP (mmHg)		121 ± 16	123 ± 17	0.09‡	116 ± 19	118 ± 21	0.49‡
Diastolic BP (mmHg)		77 ± 10	79 ± 11	**0.03** ‡	74 ± 10	74 ± 12	0.80‡
TG (mg/dL)		121 ± 81	129 ± 63	0.31‡	91 ± 66	94 ± 44	0.79‡
HDL-C (mg/dL)		52.5 ± 12.8	52.2 ± 14.0	0.81‡	64.3 ± 14.6	62.6 ± 15.3	0.56‡
LDL-C (mg/dL)		114 ± 28	119 ± 29	0.07‡	118 ± 30	125 ± 30	0.25‡
Hb-A1c (%)		5.7 ± 0.7	5.7 ± 0.7	0.93‡	5.6 ± 0.4	5.7 ± 0.4	0.42‡
HOMA-R		1.7 ± 1.4	2.0 ± 1.4	0.06‡	1.3 ± 1.0	1.3 ± 0.9	0.97‡
UA (mg/dL)		6.2 ± 1.2	6.3 ± 1.2	0.84‡	4.8 ± 1.0	5.1 ± 1.0	0.13‡
Smoking habit	Non-smoker	483 (32%)	25 (23%)	0.07†	765 (80%)	21 (75%)	0.71†
	Ex-smoker	788 (52%)	64 (58%)		138 (15%)	5 (18%)	
	Current smoking < 20 /day	105 (7%)	5 (5%)		38 (4%)	2 (7%)	
	Current smoking 20–39 /day	121 (8%)	15 (14%)		14 (2%)	0 (0%)	
	Current smoking ≥ 40 /day	17 (1%)	2 (2%)		0 (0%)	0 (0%)	
Quantity of alcohol consumption	< 40g ethanol/day	1057 (70%)	63 (57%)	**0.01** ¶	892 (93%)	26 (93%)	0.71¶
	≥ 40g ethanol/day	457 (30%)	48 (43%)		63 (7%)	2 (7%)	
Frequency of alcohol drinking	None or social	432 (29%)	23 (21%)	**0.046** †	606 (64%)	17 (61%)	0.51†
	1–4 days /week	429 (28%)	27 (24%)		196 (21%)	8 (29%)	
	5–7 days / week	653 (43%)	61 (55%)		153 (16%)	3 (11%)	
Exercise	Sweating for 30 mins twice a week	631 (42%)	43 (39%)	0.62¶	313 (33%)	8 (29%)	0.84¶
	≥ 1 hour walk / day	537 (36%)	33 (30%)	0.26¶	311 (33%)	8 (29%)	0.84¶
Eating habit	No breakfast ≥ 3 times / week	285 (19%)	21 (19%)	1.00¶	136 (14%)	3 (11%)	0.79¶
	Late evening snack ≥ 3 times / week	183 (12%)	14 (13%)	0.88¶	147 (15%)	6 (21%)	0.42¶
	Late dinner ≥ 3 times / week	370 (24%)	29 (26%)	0.73¶	152 (16%)	6 (21%)	0.43¶
Medication	Proton pump inhibitor	190 (13%)	22 (20%)	**0.04** ¶	95 (10%)	3 (11%)	0.75¶
	Aspirin	147 (10%)	7 (6%)	0.31¶	34 (4%)	1 (4%)	1.00¶
	Statin	283 (19%)	27 (24%)	0.17¶	182 (19%)	7 (25%)	0.46¶
Sleeping < 6 hours/night		852 (56%)	72 (65%)	0.09¶	558 (58%)	22 (79%)	**0.03** ¶
Sleep apnea syndrome		57 (4%)	5 (5%)	0.61¶	5 (1%)	0 (0%)	1.00¶
Hiatus hernia		503 (33%)	86 (78%)	**<0.001** ¶	219 (23%)	18 (64%)	**<0.001** ¶
Reflux esophagitis		153 (10%)	29 (26%)	**<0.001** ¶	29 (3%)	5 (18%)	**0.002** ¶

Bold values indicate significant differences. SSBE, short-segment Barrett’s esophagus; BMI, body mass index; BW, body weight; BP, blood pressure; TG, triglyceride; HDL-C, high-density lipoprotein cholesterol; LDL-C, low-density lipoprotein cholesterol; Hb-A1c, hemoglobin A1c; HOMA-R, homeostatic model assessment of insulin resistance; UA, uric acid;

^¶^, Fisher’s exact test;

^†^, χ^2^ test;

^‡^, Student’s t-test.

### Independently associated factors for the presence or the severity of reflux esophagitis or the presence of Barrett’s esophagus

Using age-adjusted logistic regression analyses and subsequent multivariable logistic regression analyses, we could identify independently associated factors for the presence or the severity of RE or the presence of BE in men (Table 4) and women (Table 5) respectively. In men, levels of VFA (odds ratio [OR], 1.21 per 50 cm^2^; 95% confident interval [CI], 1.00 to 1.46), levels of TG (OR, 1.12 per 50 mg/dL; 95% CI, 1.01 to 1.23), current smoking (OR, 1.66; 95% CI, 1.10 to 2.50), and the presence of hiatus hernia (OR, 9.29; 95% CI, 6.31 to 13.7) were independently associated with the presence of RE. More than 40g ethanol of alcohol consumption on a drinking day (OR, 2.13; 95% CI, 1.03 to 4.41), the presence of hiatus hernia (OR, 4.45; 95% CI, 1.25 to 15.9), and the presence of BE (OR, 2.66; 95% CI, 1.13 to 6.27) were independently associated with the severity of RE. More than 40g ethanol of alcohol consumption on a drinking day (OR, 1.71; 95% CI, 1.14 to 2.56) and the presence of hiatus hernia (OR, 6.86; 95% CI, 4.34 to 10.9) were also independently associated with the presence of BE. In women, levels of VFA (OR, 2.31 per 50 cm^2^; 95% CI, 1.57 to 3.40), the presence of hiatus hernia (OR, 8.98; 95% CI, 3.76 to 21.5), and the presence of BE (OR, 3.77; 95% CI, 1.20 to 11.8) were independently associated with the presence of RE. In terms of the severity of RE, no factors could be extracted in women. Sleeping less than 6 hours a night (OR, 2.95; 95% CI, 1.17 to 7.47), the presence of hiatus hernia (OR, 5.44; 95% CI, 2.38 to 12.4), and the presence of RE (OR, 3.13; 95% CI, 1.04 to 9.42) were independently associated with the presence of BE.

**Table 4:** Factors associated with reflux esophagitis or Barrett’s esophagus in men.

		Age-adjusted analysis¶		Multivariable analysis†	
		OR	(95% CI)	OR	(95% CI)
Reflux esophagitis (presence vs. absence)	BMI (per 5 kg/m^2^)	**1.45**	(1.14–1.84)		
	Waist circumference (per 5 cm)	**1.16**	(1.07–1.27)		
	Visceral fat area (per 50 cm^2^)	**1.42**	(1.21–1.66)	**1.21**	(1.01–1.46)
	≥ 10kg of BW increase since 20 y/o	**1.73**	(1.26–2.39)		
	Diastolic BP (per 10 mmHg)	**1.19**	(1.02–1.39)		
	TG (per 50 mg/dL)	**1.14**	(1.06–1.23)	**1.12**	(1.01–1.23)
	HDL-C (per 20 mg/dL)	**0.77**	(0.60–0.99)		
	LDL-C (per 20 mg/dL)	1.06	(0.95–1.18)		
	Hb-A1c (per 1%)	1.01	(0.79–1.28)		
	HOMA-R (per 1)	**1.13**	(1.03–1.23)		
	UA (per 1 mg/dL)	1.12 ▲	(0.99–1.27)		
	Current smoking	**1.79**	(1.23–2.60)	**1.66**	(1.10–2.50)
	Alcohol consumption ≥ 40g /day	1.25	(0.90–1.74)		
	Sweating for 30 mins twice a week	0.86	(0.62–1.19)		
	Late dinner ≥ 3 times / week	1.31	(0.92–1.86)		
	Aspirin use	0.65	(0.35–1.19)		
	Sleeping < 6 hours/night	**1.39**	(1.00–1.93)		
	Hiatus hernia	**9.47**	(6.46–13.9)	**9.29**	(6.31–13.7)
	SSBE	**3.24**	(2.05–5.11)		
Reflux esophagitis (LA-A vs.B–D)	Visceral fat area (per 50 cm^2^)	1.38 ▲	(0.96–1.99)		
	Systolic BP (per 10 mmHg)	1.26 ▲	(1.00–1.59)		
	Diastolic BP (per 10 mmHg)	1.27	(0.93–1.73)		
	LDL-C (per 20 mg/dL)	0.84	(0.66–1.06)		
	Alcohol consumption ≥ 40g /day	2.00 ▲	(0.97–4.14)	**2.13**	(1.03–4.41)
	Statin use	0.35 ▲	(0.09–1.10)		
	Hiatus hernia	**4.42**	(1.27–15.4)	**4.45**	(1.25–15.9)
	SSBE	**3.30**	(1.43–7.62)	**2.66**	(1.13–6.27)
Barrett’s esophagus (presence vs absence)	BMI (per 5 kg/m^2^)	1.31 ▲	(0.97–1.78)		
	Visceral fat area (per 50 cm^2^)	1.18 ▲	(0.97–1.44)		
	Systolic BP (per 10 mmHg)	1.10	(0.98–1.24)		
	Diastolic BP (per 10 mmHg)	**1.26**	(1.04–1.53)		
	LDL-C (per 20 mg/dL)	1.14 ▲	(1.00–1.31)		
	HOMA-R (per 1)	1.10 ▲	(1.00–1.22)		
	Current smoking	1.37	(0.83–2.26)		
	Alcohol consumption ≥ 40g /day	**1.91**	(1.09–2.36)	**1.71**	(1.14–2.56)
	Alcohol drinking habit ≥5 days/week	**1.60**	(1.09–2.37)		
	Proton pump inhibitor use	**1.93**	(1.10–3.38)		
	Statin use	1.40	(0.90–2.20)		
	Sleeping < 6 hours/night	**1.60**	(1.09–2.37)		
	Hiatus hernia	**6.91**	(4.38–10.9)	**6.86**	(4.34–10.9)
	Reflux esophagitis	**3.22**	(2.04–5.09)		

Bold values indicate significant differences. OR, odds ratio; CI, confident interval; BMI, body mass index; BW, body weight; BP, blood pressure; TG, triglyceride; HDL-C, high-density lipoprotein cholesterol; LDL-C, low-density lipoprotein cholesterol; Hb-A1c, hemoglobin A1c; HOMA-R, homeostatic model assessment of insulin resistance; UA, uric acid; SSBE, short-segment Barrett’s esophagus;

^▲^, p < 0.1;

^¶^, logistic regression analysis adjusted for age,

^†^, logistic regression analysis adjusted for factors selected by stepwise method form marginally associated factors in age-adjusted analyses (p < 0.1).

**Table 5:** Factors associated with reflux esophagitis or Barrett’s esophagus in women.

		Age-adjusted analysis¶		Multivariable analysis†	
		OR	(95% CI)	OR	(95% CI)
Reflux esophagitis (presence vs. absence)	BMI (per 5 kg/m^2^)	**2.12**	(1.43–3.16)		
	Waist circumference (per 5 cm)	**1.37**	(1.17–1.61)		
	Visceral fat area (per 50 cm^2^)	**1.47**	(1.27–1.71)	**2.31**	(1.57–3.40)
	≥ 10kg of BW increase since 20 y/o	**2.34**	(1.17–4.68)		
	Systolic BP (per 10 mmHg)	1.15	(0.96–1.38)		
	Diastolic BP (per 10 mmHg)	1.27	(0.92–1.76)		
	TG (per 50 mg/dL)	**1.08**	(1.02–1.14)		
	HDL-C (per 20 mg/dL)	**0.58**	(0.35–0.96)		
	LDL-C (per 20 mg/dL)	**1.24**	(1.01–1.53)		
	Hb-A1c (per 1%)	1.71 ^▲^	(0.92–3.18)		
	HOMA-R (per 1)	**1.35**	(1.07–1.69)		
	Sweating for 30 mins twice a week	0.45 ^▲^	(0.19–1.06)		
	No breakfast ≥ 3 times / week	0.43	(0.10–1.85)		
	Statin use	1.79	(0.84–3.82)		
	Hiatus hernia	**13.8**	(5.72–33.3)	**8.98**	(3.76–21.5)
	SSBE	**6.65**	(2.35–18.8)	**3.77**	(1.20–11.8)
Reflux esophagitis (LA-A vs.B–D)	Waist circumference (per 5 cm)	0.77	(0.51–1.16)		
	Hiatus hernia	N. A.			
Barrett’s esophagus (presence vs absence)	UA (per 1 mg/dL)	1.28	(0.90–1.81)		
	Sleeping < 6 hours/night	**2.70**	(1.08–6.75)	**2.95**	(1.17–7.47)
	Hiatus hernia	**6.32**	(2.75–14.5)	**5.44**	(2.38–12.4)
	Reflux esophagitis	**6.64**	(2.35–18.8)	**3.13**	(1.04–9.42)

Bold values indicate significant differences. OR, odds ratio; CI, confident interval; BMI, body mass index; BW, body weight; BP, blood pressure; TG, triglyceride; HDL-C, high-density lipoprotein cholesterol; LDL-C, low-density lipoprotein cholesterol; Hb-A1c, hemoglobin A1c; HOMA-R, homeostatic model assessment of insulin resistance; SSBE, short-segment Barrett’s esophagus;

^▲^, p < 0.1;

^¶^, logistic regression analysis adjusted for age,

^†^, logistic regression analysis adjusted for factors selected by stepwise method form marginally associated factors in age-adjusted analyses (p < 0.1).

### Subgroup analysis based on visceral fat area

After the participants were subcategorized into the presence or absence of abdominal fat accumulation using the cut-off point of VFA at 100 cm^2^, independently associated factors for the presence or the severity of reflux esophagitis or the presence of Barrett’s esophagus were also evaluated (Table 6). The results of univariable analyses and age-adjusted logistic regression analyses were shown in S1 File. In men without visceral fat accumulation, current smoking (OR, 3.10; 95% CI, 1.71 to 5.61), the presence of hiatus hernia (OR, 8.34; 95% CI, 4.59 to 15.2), and the presence of SSBE (OR, 2.27; 95% CI, 1.06 to 4.90) were independently associated with the presence of RE. Frequent alcohol intake defined as drinking 5 or more days per week (OR, 3.38; 95% CI, 1.04 to 11.0) was associated with the severity of RE. More than 40g ethanol of alcohol consumption on a drinking day (OR, 1.92; 95% CI, 1.02 to 3.62), the presence of hiatus hernia (OR, 6.40; 95% CI, 3.11 to 13.2), and the presence of reflux esophagitis (OR, 2.20; 95% CI, 1.03 to 4.68) were independently associated with the presence of BE. In men with visceral fat accumulation, levels of TG (OR, 1.15 per 50 mg/dL; 95% CI, 1.03 to 1.27), sleeping less than 6 hours a night (OR, 1.79; 95% CI, 1.15 to 2.80), and the presence of hiatus hernia (OR, 9.32; 95% CI, 5.58 to 15.6) were independently associated with the presence of RE. Levels of VFA (OR, 2.20 per 50 cm^2^; 95% CI, 1.14 to 4.25) and the presence of SSBE (OR, 4.36; 95% CI, 1.36 to 14.0) was associated with the severity of RE. The presence of hiatus hernia (OR, 9.32; 95% CI, 5.58 to 15.6) was associated with the presence of BE. In women without visceral fat accumulation, levels of VFA (OR, 3.58 per 50 cm^2^; 95% CI, 1.09 to 11.7), the presence of hiatus hernia (OR, 9.92; 95% CI, 3.11 to 31.6), and the presence of SSBE (OR, 5.48; 95% CI, 1.29 to 23.2) were independently associated with the presence of RE. The presence of hiatus hernia (OR, 4.25; 95% CI, 1.73 to 10.4) and the presence of RE (OR, 4.17; 95% CI, 1.03 to 16.9) were independently associated with the presence of BE. In women with visceral fat accumulation, the presence of hiatus hernia (OR, 9.32; 95% CI, 5.58 to 15.6) was associated with the presence of RE. Factors associated with the severity of RE in women with or without visceral fat accumulation, and factors associated with the presence of BE in women with visceral fat accumulation could not be identified.

**Table 6:** Associated factors for reflux esophagitis or Barrett’s esophagus in obese and non-obese subgroups: multivariable analysis^†^.

	Visceral fat area <100 cm^2^		Visceral fat area ≥100 cm^2^	
	Factors	OR (95% CI)	Factors	OR (95% CI)
***Men***				
Reflux esophagitis (presence vs. absence)	Current smoking	3.10 (1.71–5.61)	TG (per 50 mg/dL)	1.15 (1.03–1.27)
	Hiatus hernia	8.34 (4.59–15.2)	Sleeping < 6 hours/night	1.79 (1.15–2.80)
	SSBE	2.27 (1.06–4.90)	Hiatus hernia	9.32 (5.58–15.6)
Reflux esophagitis (LA-A vs.B–D)	Alcohol drinking habit ≥5 days/week	3.38 (1.04–11.0)	Visceral fat area (per 50 cm^2^)	2.20 (1.14–4.25)
			SSBE	4.36 (1.36–14.0)
Barrett’s esophagus (presence vs absence)	Alcohol consumption ≥ 40g /day	1.92 (1.02–3.62)	Hiatus hernia	6.17 (3.35–11.4)
	Hiatus hernia	6.40 (3.11–13.2)		
	Reflux esophagitis	2.20 (1.03–4.68)		
***Women***				
Reflux esophagitis (presence vs. absence)	Visceral fat area (per 50 cm^2^)	3.58 (1.09–11.7)	Hiatus hernia	7.16 (1.97–26.0)
	Hiatus hernia	9.92 (3.11–31.6)		
	SSBE	5.48 (1.29–23.2)		
Reflux esophagitis (LA-A vs.B–D)	N. D.		N. D.	
Barrett’s esophagus (presence vs absence)	Hiatus hernia	4.25 (1.73–10.4)	N. D.	
	Reflux esophagitis	4.17 (1.03–16.9)		

OR, odds ratio; CI, confident interval; TG, triglyceride; SSBE, short-segment Barrett’s esophagus; N. D., not detected;

^†^, logistic regression analysis adjusted for factors selected by stepwise method form marginally associated factors in age-adjusted analyses (p < 0.1)

## Discussion

The present study showed that visceral fat accumulation was associated with the presence of RE in both men and women. Especially in women, increasing levels of VFA was an independent risk factor among participants without visceral fat accumulation (VFA < 100cm^2^). This result suggests that abdominal visceral adipose tissue not only mechanically disrupts the integrity of the gastroesophageal junction barrier, but also has some endocrinological effects on the esophagus. According to previous reports, serum levels of inflammatory cytokines and adipokines, such as adiponectin, leptin, IL-6, and IL-1β, were associated with the presence of RE.[13, 14] Further investigation is required to reveal the precise role of visceral fat in the pathogenesis of RE.

In contrast to RE, the present study revealed that VFA was not associated with BE in a Japanese health-checkup population. Watari et al also recently reported that obesity, assessed by BMI, did not have an independent association with the risk of BE in a Japanese population.[15] The discrepancy of risk factors for BE between Japanese and Caucasians could be influenced by the difference of the length of BE since there were no individuals with long-segment BE (LSBE) in our study. In fact, a recent report showed that the presence of LSBE was associated with BMI even in Japan.[16] However, LSBE is a rare condition in Japan. According to a previous hospital-based survey, the prevalence of LSBE was 0.2%.[17] In addition, most of esophageal adenocarcinoma in Japanese population were derived from SSBE.[18] Therefore, it would be important to clarify risk factors for the presence of SSBE in Japanese population.

In men, excessive alcohol consumption on days in which drinking occurred was associated with both the severity of RE and the presence of BE. Frequent alcohol drinking was associated with the severity of RE in men without central obesity. Several previous studies also showed that alcohol consumption was associated with an increased risk of RE.[19–21] On the other hand, recent systematic reviews showed that alcohol consumption is not a risk factor for BE or esophageal adenocarcinoma in Caucasians.[22, 23] The existence of racial differences in alcoholic metabolism between races has been well documented. For instance, the atypical aldehyde dehydrogenase 2 gene is extremely rare in Caucasians, whereas this mutant gene is widely prevalent among the Mongoloids.[24] This suggests that the association of alcohol consumption with RE and BE could be different between the Western and the Asian population. In addition, average alcohol consumption per day was usually used to evaluate the influence of alcohol drinking. However, average volume obscured effects of quantity alone and frequency alone,[25] although alcohol quantity and frequency have been differentially related to diseases including diabetes and non-fatal myocardial infarction.[26] Therefore, we evaluated the frequency and quantity of alcohol separately and only the quantity on drinking days was associated with the severity of RE and the prevalence of BE. Interestingly, in patients with non-alcoholic fatty liver disease NAFLD, VFA was reported to be associated with the presence of BE in Japan,[27] suggesting that central obesity could be a risk factor for BE in non-alcohol-drinking Japanese population.

Short duration of sleep was associated with the presence of RE in men with central obesity and the presence of BE in women. Sleep apnea syndrome was not associated with RE or BE. A recent report showed that short sleeping duration was correlated with the severity of GERD symptoms.[28] In addition, one systematic review and meta-analysis showed the protective effect of long sleep duration on hormone-related cancer, such as breast cancer and epithelial ovarian cancer. However, association between sleep duration and esophageal adenocarcinoma was not reported. Further studies are warranted.

The limitations of the present study include the relatively small sample size especially in female patients with RE or BE. This may cause our multivariable models to be overfit. Another criticism is that we did not perform histological diagnosis and could not evaluate the factors associated with the progression of BE. In Japan, pathological diagnosis of esophageal adenocarcinoma is performed using tissue samples obtained by narrow band imaging targeted biopsy. Conducting random biopsy for all individuals with columnar-lined esophagus is not thought to be cost effective especially in Japanese medical checkup population, since the incidence of esophageal adenocarcinoma is still extremely rare even in men. It would be difficult to distinguish between gastric metaplasia and intestinal metaplasia using endoscopy,[29] individuals who had columnar-lined esophagus but did not have intestinal metaplasia would be included into BE-positive group.

In conclusion, we identified that factors associated with BE were different from RE in Japanese healthy population. Levels of visceral fat area were associated with the presence of RE both in men and women, but not with the presence of BE. Excess quantity of alcohol consumption on a drinking day in men was associated with the severity of RE as well as the presence of BE. These data can provide helpful information about lifestyle habit improvement for GERD patients, and may be useful for the prevention of subsequent esophageal adenocarcinoma.

## Supporting Information

S1 File Subgroup analysis based on visceral fat area. Differences between participants with and without reflux esophagitis in non-obese and obese men (Table A). Differences between participants with and without reflux esophagitis in non-obese and obese women (Table B). Differences between participants with mild and severe reflux esophagitis in non-obese and obese men (Table C). Differences between participants with mild and severe reflux esophagitis in non-obese and obese women (Table D). Differences between participants with and without short-segment Barrett’s esophagus in non-obese and obese men (Table E). Differences between participants with and without short-segment Barrett’s esophagus in non-obese and obese women (Table F). Factors associated with reflux esophagitis or Barrett’s esophagus in non-obese men (Visceral fat area <100 cm^2^) (Table G). Factors associated with reflux esophagitis or Barrett’s esophagus in obese men (Visceral fat area ≥100 cm^2^) (Table H). Factors associated with reflux esophagitis or Barrett’s esophagus in non-obese women (Visceral fat area <100 cm^2^) (Table I). Factors associated with reflux esophagitis or Barrett’s esophagus in obese women (Visceral fat area ≥100 cm^2^) (Table J).
(DOC)

S1 Raw Data The raw data (SAV). (SAV)
