# Supplementary material for: Association of Visceral Fat Area, Smoking, and Alcohol Consumption with Reflux Esophagitis and Barrett's Esophagus in Japan
Source: PLoS One. 2015 Jul 30;10(7):e0133865. doi: 10.1371/journal.pone.0133865 (PMC4520496; doi:10.1371/journal.pone.0133865)
Supplement: S1 File — Differences between participants with and without reflux esophagitis in non-obese and obese men (Table A). Differences between participants with and without reflux esophagitis in non-obese and obese women (Table B). Differences between participants with mild and severe reflux esophagitis in non-obese and obese men (Table C). Differences between participants with mild and severe reflux esophagitis in non-obese and obese women (Table D). Differences between participants with and without short-segment Barrett’s esophagus in non-obese and obese men (Table E). Differences between participants with and without short-segment Barrett’s esophagus in non-obese and obese women (Table F). Factors associated with reflux esophagitis or Barrett’s esophagus in non-obese men (Visceral fat area <100 cm2) (Table G). Factors associated with reflux esophagitis or Barrett’s esophagus in obese men (Visceral fat area ≥100 cm2) (Table H). Factors associated with reflux esophagitis or Barrett’s esophagus in non-obese women (Visceral fat area <100 cm2) (Table I). Factors associated with reflux esophagitis or Barrett’s esophagus in obese women (Visceral fat area ≥100 cm2) (Table J). (DOC) [file pone.0133865.s001.doc]

***Table A. Differences between participants with and without reflux esophagitis in non-obese and obese men***

|  | Visceral fat area <100 cm^2^ | | | Visceral fat area ≥100 cm^2^ | | |
| --- | --- | --- | --- | --- | --- | --- |
|  | RE (-)  (n = 756) | RE (+)  (n =70) | p value | RE (-)  (n = 687) | RE (+)  (n =112) | p value |
| Age (y)  BMI (kg/m^2^)  Waist circumference (cm)  Visceral fat area (cm^2^)  ≥ 10kg of BW increase since 20 y/o  Systolic BP (mmHg)  Diastolic BP (mmHg)  TG (mg/dL)  HDL-C (mg/dL)  LDL-C (mg/dL)  Hb-A1c (%)  HOMA-R  UA (mg/dL) | 58.8 ± 13.6  22.7 ± 2.3  78.8 ± 6.3  68 ± 22  248 (33 %)  118 ± 17  76 ± 10  100 ± 53  55.8 ± 13.2  112 ± 27  5.6 ± 0.6  1.3 ± 0.9  6.1 ± 1.2 | 53.5 ± 12.9  23.1 ± 1.8  79.7 ± 5.2  73 ± 21  26 (37 %)  116 ± 12  75 ± 9  112 ± 56  54.3 ± 14.8  115 ± 30  5.6 ± 0.5  1.4 ± 0.8  6.0 ± 1.3 | **0.002‡**  0.07‡  0.27‡  0.12‡  0.50 ¶  0.24‡  0.49‡  0.08‡  0.37‡  0.37‡  0.57‡  0.22‡  0.92‡ | 63.0 ± 11.8  25.4 ± 3.0  88.3 ± 7.6  142 ± 36  481 (70 %)  124 ± 15  78 ± 10  139 ± 88  49.4 ± 11.4  116 ± 29  5.9 ± 0.7  2.1 ± 1.7  6.4 ± 1.2 | 60.8 ± 11.7  25.8 ± 2.8  89.5 ± 6.6  145 ± 37  91 (81 %)  125 ± 16  81 ± 11  164 ± 136  48.2 ± 12.0  118 ± 30  5.8 ± 0.7  2.4 ± 1.6  6.7 ± 1.2 | 0.08‡  0.18‡  0.12‡  0.36‡  **0.017 ¶**  0.32‡  **0.002‡**  0.06‡  0.28‡  0.51‡  0.22‡  0.09‡  0.05‡ |
| Smoking habit  Non-smoker  Ex-smoker  Current smoker  < 20 /day  20-39 /day  ≥ 40 /day  Quantity of alcohol consumption  < 40g ethanol/day  ≥ 40g ethanol/day  Frequency of alcohol drinking  None or social  1 – 4 days /week  5 – 7 days / week | 279 (37 %)  372 (49 %)  55 (7 %)  48 (6 %)  2 (0.3 %)  544 (72 %)  212 (28 %)  230 (30 %)  214 (28 %)  312 (41 %) | 20 (29 %)  28 (40 %)  5 (7 %)  13 (19 %)  4 (6 %)  48 (69 %)  22 (31 %)  20 (29 %)  16 (23 %)  34 (49 %) | **<0.001**†  0.58 ¶  0.46† | 182 (27 %)  392 (57 %)  38 (6 %)  64 (9 %)  11 (2 %)  462 (67 %)  225 (33 %)  179 (26 %)  196 (29 %)  312 (45 %) | 27 (24 %)  60 (54 %)  12 (11 %)  11 (10 %)  2 (2 %)  66 (59 %)  46 (41 %)  26 (23 %)  30 (27 %)  56 (50 %) | 0.33†  0.09 ¶  0.66† |
| Exercise  Sweating for 30 mins twice a week  ≥ 1 hour walk / day  Eating habit  No breakfast ≥ 3 times / week  Late evening snack ≥ 3 times / week  Late dinner ≥ 3 times / week  Medication  Proton pump inhibitor  Aspirin  Statin  Sleeping < 6 hours/night  Sleep apnea syndrome  Hiatus hernia  SSBE | 335 (44 %)  287 (38 %)  142 (19 %)  87 (12 %)  196 (26 %)  70 (9 %)  62 (8 %)  108 (14 %)  429 (57 %)  18 (2 %)  206 (27 %)  33 (4 %) | 30 (43 %)  31 (44 %)  20 (29 %)  9 (13 %)  22 (31 %)  11 (16 %)  2 (3 %)  9 (13 %)  44 (63 %)  2 (3 %)  54 (77 %)  12 (17 %) | 0.90¶  0.31¶  0.06¶  0.70¶  0.32¶  0.09¶  0.16¶  0.86¶  **0.01**¶  0.68¶  **<0.001¶**  **<0.001**¶ | 272 (40 %)  220 (32 %)  123 (18 %)  84 (12 %)  146 (21 %)  116 (17 %)  80 (12 %)  173 (25 %)  376 (55 %)  36 (5 %)  238 (35 %)  49 (7 %) | 37 (33 %)  32 (29 %)  21 (19 %)  17 (15 %)  35 (31 %)  15 (13 %)  10 (9 %)  20 (18 %)  75 (67 %)  6 (5 %)  91 (81 %)  17 (15 %) | 0.21¶  0.51¶  0.79¶  0.36¶  **0.03**¶  0.41¶  0.52¶  0.10¶  **0.01**¶  1.00¶  **<0.001**¶  **0.008**¶ |

Bold values indicate significant differences. RE, reflux esophagitis; BMI, body mass index; BW, body weight; BP, blood pressure; TG, triglyceride; HDL-C, high-density lipoprotein cholesterol; LDL-C, low-density lipoprotein cholesterol; Hb-A1c, hemoglobin A1c; HOMA-R, homeostatic model assessment of insulin resistance; UA, uric acid; SSBE, short-segment Barrett’s esophagus; ¶, Fisher’s exact test; †, χ^2^ test; ‡, Student’s t-test.

***Table B. Differences between participants with and without reflux esophagitis in non-obese and obese women***

|  | Visceral fat area <100 cm^2^ | | | Visceral fat area ≥100 cm^2^ | | |
| --- | --- | --- | --- | --- | --- | --- |
|  | RE (-)  (n =802) | RE (+)  (n =17) | p value | RE (-)  (n =147) | RE (+)  (n =17) | p value |
| Age (y)  BMI (kg/m^2^)  Waist circumference (cm)  Visceral fat area (cm^2^)  ≥ 10kg of BW increase since 20 y/o  Systolic BP (mmHg)  Diastolic BP (mmHg)  TG (mg/dL)  HDL-C (mg/dL)  LDL-C (mg/dL)  Hb-A1c (%)  HOMA-R  UA (mg/dL) | 57.2 ± 13.3  20.8 ± 2.6  76.0 ± 8.5  52 ± 24  150 (19 %)  113 ± 18  73 ± 10  82 ± 53  66.1 ± 14.4  117 ± 30  5.5 ± 0.4  1.1 ± 1.2  4.7 ± 1.0 | 62.4 ± 11.4  21.7 ± 2.8  78.2 ± 8.5  68 ± 19  3 (18 %)  121 ± 17  75 ± 12  100 ± 43  63.2 ± 12.1  127 ± 22  5.7 ± 0.4  1.2 ± 0.5  4.7 ± 0.7 | 0.11‡  0.14‡  0.29‡  **0.005‡**  1.00¶  0.07‡  0.37‡  0.17‡  0.41‡  0.17‡  0.19‡  0.55‡  0.78‡ | 66.6 ± 10.7  25.8 ± 3.7  90.7 ± 8.6  130 ± 30  101 (69 %)  128 ± 19  77 ± 11  131 ± 94  55.0 ± 11.7  120 ± 31  5.9 ± 0.5  2.4 ± 1.6  5.4 ± 1.1 | 63.6 ± 9.7  26.1 ± 4.0  93.5 ± 7.4  142 ± 32  13 (77 %)  125 ± 15  78 ± 8  149 ± 111  54.6 ± 13.6  132 ± 49  5.9 ± 0.7  2.4 ± 1.6  5.1 ± 1.0 | 0.27‡  0.74‡  0.195‡  0.12‡  0.59¶  0.58‡  0.74‡  0.48‡  0.90‡  0.16‡  0.88‡  0.96‡  0.38‡ |
| Smoking habit  Non-smoker  Ex-smoker  Current smoker  < 20 /day  20-39 /day  Quantity of alcohol consumption  < 40g ethanol/day  ≥ 40g ethanol/day  Frequency of alcohol drinking  None or social  1 – 4 days /week  5 – 7 days / week | 641 (80 %)  117 (15 %)  33 (4 %)  11 (1 %)  746 (93 %)  56 (7 %)  486 (61 %)  175 (22 %)  141 (18 %) | 20 (29 %)  28 (40 %)  5 (7 %)  13 (19 %)  16 (94 %)  1 (6 %)  12 (71 %)  3 (18 %)  2 (12 %) | 0.93†  1.00 ¶  0.69† | 118 (80 %)  21 (14 %)  6 (5 %)  2 (1 %)  141 (96 %)  6 (4 %)  113 (77 %)  24 (16 %)  10 (7 %) | 13 (77 %)  3 (18 %)  0 (0 %)  1 (6 %)  15 (88 %)  2 (12 %)  12 (71 %)  2 (12 %)  3 (18 %) | 0.47†  0.195 ¶  0.28† |
| Exercise  Sweating for 30 mins twice a week  ≥ 1 hour walk / day  Eating habit  No breakfast ≥ 3 times / week  Late evening snack ≥ 3 times / week  Late dinner ≥ 3 times / week  Medication  Proton pump inhibitor  Aspirin  Statin  Sleeping < 6 hours/night  Sleep apnea syndrome  Hiatus hernia  SSBE | 269 (34 %)  256 (32 %)  120 (15 %)  121 (15 %)  15 (10 %)  63 (8 %)  23 (3 %)  125 (16 %)  478 (60 %)  4 (1 %)  152 (19 %)  19 (2 %) | 4 (24 %)  9 (53 %)  0 (0 %)  2 (12 %)  3 (18 %)  2 (12 %)  0 (0 %)  3 (18 %)  8 (47 %)  0 (0 %)  13 (77 %)  3 (18 %) | 0.45¶  0.11¶  0.16¶  1.00¶  0.51¶  0.64¶  1.00¶  0.74¶  0.33¶  1.00¶  **<0.001¶**  **0.009**¶ | 45 (31 %)  51 (35 %)  17 (12 %)  25 (17 %)  151 (16 %)  32 (21 %)  11 (8 %)  54 (37 %)  81 (55 %)  1 (1 %)  58 (40 %)  4 (3 %) | 3 (18 %)  3 (18 %)  2 (12 %)  5 (29 %)  7 (21 %)  2 (12 %)  1 (6 %)  7 (41 %)  13 (77 %)  0 (0 %)  14 (82 %)  2 (12 %) | 0.40¶  0.18¶  1.00¶  0.20¶  0.41¶  0.53¶  1.00¶  0.79¶  0.12¶  1.00¶  **0.001**¶  0.12¶ |

Bold values indicate significant differences. RE, reflux esophagitis; BMI, body mass index; BW, body weight; BP, blood pressure; TG, triglyceride; HDL-C, high-density lipoprotein cholesterol; LDL-C, low-density lipoprotein cholesterol; Hb-A1c, hemoglobin A1c; HOMA-R, homeostatic model assessment of insulin resistance; UA, uric acid; SSBE, short-segment Barrett’s esophagus; ¶, Fisher’s exact test; †, χ^2^ test; ‡, Student’s t-test.

***Table C. Differences between participants with mild and severe reflux esophagitis in non-obese and obese men***

|  | Visceral fat area <100 cm^2^ | | | Visceral fat area ≥100 cm^2^ | | |
| --- | --- | --- | --- | --- | --- | --- |
|  | RE, A  (n = 53) | RE, B-D  (n =17) | p value | RE, A  (n = 85) | RE, B-D  (n =27) | p value |
| Age (y)  BMI (kg/m^2^)  Waist circumference (cm)  Visceral fat area (cm^2^)  ≥ 10kg of BW increase since 20 y/o  Systolic BP (mmHg)  Diastolic BP (mmHg)  TG (mg/dL)  HDL-C (mg/dL)  LDL-C (mg/dL)  Hb-A1c (%)  HOMA-R  UA (mg/dL) | 52.4 ± 11.4  23.3 ± 1.9  80.1 ± 5.6  71 ± 21  21 (40 %)  116 ± 12  75 ± 9  109 ± 52  54.2 ± 15.8  118 ± 30  5.6 ± 0.5  1.5 ± 0.8  5.9 ± 1.4 | 56.9 ± 16.8  22.4 ± 1.1  78.3 ± 3.7  77 ± 19  5 (29 %)  118 ± 10  74 ± 11  123 ± 68  54.5 ± 11.6  104 ± 28  5.5 ± 0.5  1.3 ± 0.6  6.4 ± 0.9 | 0.32‡  0.08‡  0.15‡  0.32‡  0.57¶  0.64‡  0.72‡  0.38‡  0.94‡  0.10‡  0.63‡  0.43‡  0.08‡ | 61.0 ± 11.9  25.4 ± 2.6  88.5 ± 5.9  140 ± 30  68 (80 %)  123 ± 16  80 ± 11  164 ± 152  48.3 ± 11.2  119 ± 30  5.8 ± 0.8  2.3 ± 1.6  6.6 ± 1.2 | 60.4 ± 11.0  27.1 ± 2.9  92.7 ± 7.6  161 ± 50  23 (85 %)  131 ± 17  85 ± 10  163 ± 67  47.6 ± 14.3  115 ± 32  5.8 ± 0.5  2.7 ± 1.5  6.7 ± 1.3 | 0.84‡  **0.005‡**  **0.003‡**  **0.04‡**  0.78¶  **0.03‡**  **0.04‡**  0.96‡  0.79‡  0.52‡  0.79‡  0.21‡  0.75‡ |
| Smoking habit  Non-smoker  Ex-smoker  Current smoker  < 20 /day  20-39 /day  ≥ 40 /day  Quantity of alcohol consumption  < 40g ethanol/day  ≥ 40g ethanol/day  Frequency of alcohol drinking  None or social  1 – 4 days /week  5 – 7 days / week | 16 (30 %)  22 (42 %)  4 (8 %)  10 (19 %)  1 (2 %)  39 (74 %)  14 (26 %)  17 (32 %)  14 (26 %)  22 (42 %) | 4 (24 %)  6 (35 %)  1 (6 %)  3 (18 %)  3 (18 %)  9 (53 %)  8 (47 %)  3 (18 %)  2 (12 %)  12 (71 %) | 0.20†  0.14¶  0.11† | 22 (26 %)  44 (52 %)  9 (11 %)  8 (9 %)  2 (2 %)  52 (61 %)  33 (39 %)  21 (25 %)  21 (25 %)  43 (51 %) | 5 (19 %)  16 (59 %)  3 (11 %)  3 (11 %)  0 (0 %)  14 (52 %)  13 (48 %)  5 (19 %)  9 (33 %)  14 (48 %) | 0.85†  0.50¶  0.63† |
| Exercise  Sweating for 30 mins twice a week  ≥ 1 hour walk / day  Eating habit  No breakfast ≥ 3 times / week  Late evening snack ≥ 3 times / week  Late dinner ≥ 3 times / week  Medication  Proton pump inhibitor  Aspirin  Statin  Sleeping < 6 hours/night  Sleep apnea syndrome  Hiatus hernia  SSBE | 25 (47 %)  23 (43 %)  14 (26 %)  6 (11 %)  15 (28 %)  6 (11 %)  2 (4 %)  8 (15 %)  33 (62 %)  2 (4 %)  39 (74 %)  8 (15 %) | 5 (29 %)  8 (47 %)  6 (35 %)  3 (18 %)  7 (41 %)  5 (29 %)  0 (0 %)  1 (6 %)  11 (65 %)  0 (0 %)  15 (88 %)  4 (24 %) | 0.26¶  1.00¶  0.68¶  1.00¶  0.37¶  0.12¶  1.00¶  0.44¶  1.00¶  1.00¶  0.32¶  0.47¶ | 29 (34 %)  28 (33 %)  17 (20 %)  14 (17 %)  25 (29 %)  12 (14 %)  8 (9 %)  18 (21 %)  57 (67 %)  4 (5 %)  65 (77 %)  8 (9 %) | 8 (30 %)  4 (15 %)  4 (15 %)  3 (11 %)  10 (37 %)  3 (11 %)  2 (7 %)  2 (7 %)  18 (67 %)  2 (7 %)  26 (96 %)  9 (33 %) | 0.82¶  0.09¶  0.76¶  1.00¶  0.48¶  1.00¶  1.00¶  0.15¶  1.00¶  0.63¶  **0.02¶**  **0.005**¶ |

Bold values indicate significant differences. RE, reflux esophagitis; BMI, body mass index; BW, body weight; BP, blood pressure; TG, triglyceride; HDL-C, high-density lipoprotein cholesterol; LDL-C, low-density lipoprotein cholesterol; Hb-A1c, hemoglobin A1c; HOMA-R, homeostatic model assessment of insulin resistance; UA, uric acid; SSBE, short-segment Barrett’s esophagus; ¶, Fisher’s exact test: †, χ^2^ test: ‡, Student’s t-test.

***Table D. Differences between participants with mild and severe reflux esophagitis in non-obese and obese women***

|  | Visceral fat area <100 cm^2^ | | | Visceral fat area ≥100 cm^2^ | | |
| --- | --- | --- | --- | --- | --- | --- |
|  | RE, A  (n = 12) | RE, B-D  (n = 5) | p value | RE, A  (n = 14) | RE, B-D  (n = 3) | p value |
| Age (y)  BMI (kg/m^2^)  Waist circumference (cm)  Visceral fat area (cm^2^)  ≥ 10kg of BW increase since 20 y/o  Systolic BP (mmHg)  Diastolic BP (mmHg)  TG (mg/dL)  HDL-C (mg/dL)  LDL-C (mg/dL)  Hb-A1c (%)  HOMA-R  UA (mg/dL) | 58.8 ± 9.4  21.6 ± 2.3  78.3 ± 9.3  67 ± 23  1 (8 %)  123 ± 16  75 ± 11  100 ± 47  64.3 ± 13.7  130 ± 19  5.6 ± 0.4  1.2 ± 0.6  4.6 ± 0.9 | 71.0 ± 12.0  22.1 ± 4.0  78.1 ± 7.4  71 ± 6  2 (40 %)  117 ± 19  74 ± 14  100 ± 34  60.6 ± 7.2  122 ± 29  5.9 ± 0.3  1.3 ± 0.2  5.1 ± 0.2 | **0.04‡**  0.79‡  0.97‡  0.57‡  0.19¶  0.48‡  0.86‡  0.99‡  0.58‡  0.54‡  0.13‡  0.61‡  0.10‡ | 63.9 ± 9.2  26.4 ± 4.3  95.0 ± 7.3  142 ± 36  11 (79 %)  125 ± 16  78 ± 9  149 ± 119  54.5 ± 14.5  124 ± 26  5.9 ± 0.8  2.3 ± 1.5  5.1 ± 1.0 | 62.0 ± 14.0  24.6 ± 1.7  86.4 ± 1.6  141 ± 11  2 (67 %)  125 ± 10  78 ± 4  149 ± 78  55.3 ± 11.0  171 ± 108  5.8 ± 0.3  2.8 ± 1.9  5.4 ± 1.1 | 0.76‡  0.48‡  **0.001‡**  0.93‡  1.00¶  0.98‡  0.96‡  0.99‡  0.93‡  0.53‡  0.82‡  0.66‡  0.60‡ |
| Smoking habit  Non-smoker  Ex-smoker  Current smoker  < 20 /day  20-39 /day  Quantity of alcohol consumption  < 40g ethanol/day  ≥ 40g ethanol/day  Frequency of alcohol drinking  None or social  1 – 4 days /week  5 – 7 days / week | 10 (83 %)  1 (8 %)  1 (8 %)  0 (0 %)  11 (92 %)  1 (8 %)  8 (67 %)  3 (25 %)  1 (8 %) | 4 (80 %)  1 (20 %)  0 (0 %)  0 (0 %)  5 (100 %)  0 (0 %)  4 (80 %)  0 (0 %)  1 (20 %) | 0.66†  1.00¶  0.42† | 10 (71 %)  2 (21 %)  0 (0 %)  1 (7 %)  13 (93 %)  1 (7 %)  10 (71 %)  2 (14 %)  2 (14 %) | 3 (100 %)  0 (0 %)  0 (0 %)  0 (0 %)  2 (67 %)  1 (33 %)  2 (67 %)  0 (0 %)  1 (33 %) | 0.57†  0.33¶  0.62† |
| Exercise  Sweating for 30 mins twice a week  ≥ 1 hour walk / day  Eating habit  No breakfast ≥ 3 times / week  Late evening snack ≥ 3 times / week  Late dinner ≥ 3 times / week  Medication  Proton pump inhibitor  Aspirin  Statin  Sleeping < 6 hours/night  Sleep apnea syndrome  Hiatus hernia  SSBE | 1 (8 %)  7 (58 %)  0 (0 %)  2 (17 %)  2 (17 %)  1 (8 %)  0 (0 %)  1 (8 %)  7 (58 %)  0 (0 %)  8 (67 %)  2 (17 %) | 3 (60 %)  2 (40 %)  0 (0 %)  0 (0 %)  2 (40 %)  1 (20 %)  0 (0 %)  2 (40 %)  1 (20 %)  0 (0 %)  5 (100 %)  1 (20 %) | 0.05¶  0.62¶  1.00¶  1.00¶  0.54¶  0.52¶  1.00¶  0.19¶  0.29¶  N. A.  0.26¶  1.00¶ | 3 (21 %)  3 (21 %)  2 (14 %)  4 (29 %)  3 (21 %)  2 (14 %)  1 (7 %)  7 (50 %)  11 (79 %)  0 (0 %)  11 (79 %)  1 (7 %) | 0 (0 %)  0 (0 %)  0 (0 %)  1 (33 %)  0 (0 %)  0 (0 %)  0 (0 %)  0 (0 %)  2 (67 %)  0 (0 %)  3 (100 %)  1 (33 %) | 1.00¶  1.00¶  1.00¶  1.00¶  1.00¶  1.00¶  1.00¶  0.23¶  1.00¶  N. A.  1.00¶  0.33¶ |

Bold values indicate significant differences. RE, reflux esophagitis; BMI, body mass index; BW, body weight; BP, blood pressure; TG, triglyceride; HDL-C, high-density lipoprotein cholesterol; LDL-C, low-density lipoprotein cholesterol; Hb-A1c, hemoglobin A1c; HOMA-R, homeostatic model assessment of insulin resistance; UA, uric acid; SSBE, short-segment Barrett’s esophagus; ¶, Fisher’s exact test: †, χ^2^ test: ‡, Student’s t-test.

***Table E. Differences between participants with and without short-segment Barrett’s esophagus in non-obese and obese men***

|  | Visceral fat area <100 cm^2^ | | | Visceral fat area ≥100 cm^2^ | | |
| --- | --- | --- | --- | --- | --- | --- |
|  | SSBE (-)  (n = 781) | SSBE (+)  (n =45) | p value | SSBE (-)  (n =733) | SSBE (+)  (n =66) | p value |
| Age (y)  BMI (kg/m^2^)  Waist circumference (cm)  Visceral fat area (cm^2^)  ≥ 10kg of BW increase since 20 y/o  Systolic BP (mmHg)  Diastolic BP (mmHg)  TG (mg/dL)  HDL-C (mg/dL)  LDL-C (mg/dL)  Hb-A1c (%)  HOMA-R  UA (mg/dL) | 58.2 ± 13.7  22.7 ± 2.3  78.9 ± 6.3  68 ± 22  260 (33 %)  118 ± 16  76 ± 10  101 ± 53  55.5 ± 13.3  112 ± 27  5.6 ± 0.6  1.3 ± 0.9  6.1 ± 1.2 | 60.3 ± 11.3  22.7 ± 2.2  78.0 ± 5.7  72 ± 19  14 (31 %)  118 ± 15  77 ± 12  102 ± 60  58.5 ± 14.5  113 ± 31  5.5 ± 0.5  1.2 ± 0.7  6.1 ± 1.2 | 0.25‡  0.90‡  0.36‡  0.27‡  0.87¶  0.99‡  0.27‡  0.91‡  0.15‡  0.81‡  0.58‡  0.72‡  0.92‡ | 62.7 ± 11.9  25.4 ± 2.9  88.5 ± 7.4  142.3 ± 35  524 (72 %)  123 ± 15  79 ± 10  142 ± 99  49.4 ± 11.5  116 ± 29  5.8 ± 0.7  2.1 ± 1.7  6.5 ± 1.2 | 62.0 ± 10.7  25.7 ± 3.2  88.6 ± 8.3  140 ± 40  48 (73 %)  127 ± 17  81 ± 10  147 ± 58  48.0 ± 12.1  123 ± 26  5.8 ± 0.8  2.4 ± 1.5  6.4 ± 1.2 | 0.63‡  0.38‡  0.86‡  0.58‡  0.89¶  0.08‡  0.12‡  0.66‡  0.35‡  0.06‡  0.98‡  0.14‡  0.77‡ |
| Smoking habit  Non-smoker  Ex-smoker  Current smoker  < 20 /day  20-39 /day  ≥ 40 /day  Quantity of alcohol consumption  < 40g ethanol/day  ≥ 40g ethanol/day  Frequency of alcohol drinking  None or social  1 – 4 days /week  5 – 7 days / week | 288 (37 %)  373 (48 %)  58 (7 %)  57 (7 %)  5 (1 %)  566 (73 %)  215 (28 %)  240 (31 %)  221 (28 %)  320 (41 %) | 11 (24 %)  27 (60 %)  2 (4 %)  4 (9 %)  1 (2 %)  26 (58 %)  19 (42 %)  10 (22 %)  9 (20 %)  26 (58 %) | 0.26†  **0.04**¶  0.09† | 195 (27 %)  415 (57 %)  47 (6 %)  64 (9 %)  12 (2 %)  491 (67 %)  242 (33 %)  192 (26 %)  208 (28 %)  33 (45 %) | 14 (21 %)  37 (56 %)  3 (5 %)  11 (17 %)  1 (2 %)  37 (56 %)  29 (44 %)  13 (20 %)  18 (27 %)  35 (53 %) | 0.28†  0.08¶  0.41† |
| Exercise  Sweating for 30 mins twice a week  ≥ 1 hour walk / day  Eating habit  No breakfast ≥ 3 times / week  Late evening snack ≥ 3 times / week  Late dinner ≥ 3 times / week  Medication  Proton pump inhibitor  Aspirin  Statin  Sleeping < 6 hours/night  Sleep apnea syndrome  Hiatus hernia  Reflux esophagitis | 344 (44 %)  300 (38 %)  153 (20 %)  89 (11 %)  88 (11 %)  73 (9 %)  60 (8 %)  107 (14 %)  444 (57 %)  20 (3 %)  226 (29 %)  58 (7 %) | 21 (47 %)  18 (40 %)  9 (20 %)  7 (16 %)  8 (18 %)  8 (18 %)  4 (9 %)  10 (22 %)  29 (64 %)  0 (0 %)  34 (76 %)  12 (27 %) | 0.76¶  0.88¶  1.00¶  0.35¶  0.23¶  0.07¶  0.77¶  0.12¶  0.36¶  0.62¶  **<0.001**¶  **<0.001**¶ | 287 (39 %)  237 (32 %)  132 (18 %)  94 (13 %)  154 (21 %)  117 (16 %)  87 (12 %)  176 (24 %)  408 (56 %)  37 (5 %)  277 (38 %)  95 (13 %) | 22 (33 %)  15 (23 %)  12 (18 %)  7 (11 %)  13 (20 %)  14 (21 %)  3(5 %)  17 (26 %)  43 (65 %)  5 (8 %)  52 (79 %)  17 (26 %) | 0.43¶  0.13¶  1.00¶  0.70¶  0.88¶  0.30¶  0.10¶  0.76¶  0.16¶  0.38¶  <**0.001**¶  **0.008**¶ |

Bold values indicate significant differences. SSBE, short-segment Barrett’s esophagus;BMI, body mass index; BW, body weight; BP, blood pressure; TG, triglyceride; HDL-C, high-density lipoprotein cholesterol; LDL-C, low-density lipoprotein cholesterol; Hb-A1c, hemoglobin A1c; HOMA-R, homeostatic model assessment of insulin resistance; UA, uric acid; ¶, Fisher’s exact test: †, χ^2^ test: ‡, Student’s t-test.

***Table F. Differences between participants with and without short-segment Barrett’s esophagus in non-obese and obese women***

|  | Visceral fat area <100 cm^2^ | | | Visceral fat area ≥100 cm^2^ | | |
| --- | --- | --- | --- | --- | --- | --- |
|  | SSBE (-)  (n = 797) | SSBE (+)  (n =22) | p value | SSBE (-)  (n =158) | SSBE (+)  (n =6) | p value |
| Age (y)  BMI (kg/m^2^)  Waist circumference (cm)  Visceral fat area (cm^2^)  ≥ 10kg of BW increase since 20 y/o  Systolic BP (mmHg)  Diastolic BP (mmHg)  TG (mg/dL)  HDL-C (mg/dL)  LDL-C (mg/dL)  Hb-A1c (%)  HOMA-R  UA (mg/dL) | 57.2 ± 13.3  20.8 ± 2.6  76.1 ± 8.5  52 ± 24  150 (19 %)  113 ± 18  73 ± 10  82 ± 54  66.1 ± 14.4  117 ± 30  5.5 ± 0.4  1.1 ± 0.7  4.7 ± 1.0 | 59.1 ± 12.4  20.3 ± 1.6  75.8 ± 5.5  53 ± 18  3 (14 %)  118 ± 23  74 ± 13  89 ± 39  65.9 ± 14.2  130 ± 27  5.6 ± 0.4  1.1 ± 0.5  5.0 ± 0.9 | 0.51‡  0.12‡  0.85‡  0.85‡  0.78¶  0.27‡  0.60‡  0.55‡  0.95‡  0.054‡  0.27‡  0.74‡  0.17‡ | 66.2 ± 10.6  25.8 ± 3.8  91.2 ± 8.5  132 ± 30  110 (70 %)  128 ± 19  78 ± 11  134 ± 97  55.2 ± 11.8  122 ± 33  5.9 ± 0.5  2.4 ± 1.6  5.4 ± 1.1 | 69.3 ± 8.8  24.9 ± 1.4  85.5 ± 3.6  127 ± 17  4 (67 %)  120 ± 13  75 ± 5  112 ± 61  50.8 ± 14.2  107 ± 37  5.8 ± 0.2  2.3 ± 1.4  5.5 ± 1.3 | 0.48‡  0.53‡  **0.009‡**  0.74‡  1.00¶  0.35‡  0.48‡  0.59‡  0.38‡  0.27‡  0.61‡  0.84‡  0.72‡ |
| Smoking habit  Non-smoker  Ex-smoker  Current smoker  < 20 /day  20-39 /day  Quantity of alcohol consumption  < 40g ethanol/day  ≥ 40g ethanol/day  Frequency of alcohol drinking  None or social  1 – 4 days /week  5 – 7 days / week | 640 (80 %)  114 (14 %)  32 (4 %)  11 (1 %)  742 (93 %)  55 (7 %)  486 (61 %)  171 (22 %)  140 (18 %) | 15 (68 %)  5 (23 %)  2 (9 %)  0 (0 %)  20 (91 %)  2 (9 %)  12 (55 %)  7 (32 %)  3 (14 %) | 0.38†  0.66¶  0.50† | 125 (79 %)  24 (15 %)  6 (4 %)  3 (2 %)  150 (95 %)  8 (5 %)  120 (76 %)  25 (16 %)  13 (8 %) | 6 (100 %)  0 (0 %)  0 (0 %)  0 (0 %)  6 (100 %)  0 (0 %)  5 (83 %)  1 (17 %)  0 (0 %) | 0.67†  1.00¶  0.76† |
| Exercise  Sweating for 30 mins twice a week  ≥ 1 hour walk / day  Eating habit  No breakfast ≥ 3 times / week  Late evening snack ≥ 3 times / week  Late dinner ≥ 3 times / week  Medication  Proton pump inhibitor  Aspirin  Statin  Sleeping < 6 hours/night  Sleep apnea syndrome  Hiatus hernia  Reflux esophagitis | 266 (33 %)  258 (32 %)  117 (15 %)  118 (15 %)  134 (17 %)  62 (8 %)  23 (3 %)  124 (16 %)  470 (59 %)  4 (1 %)  153 (19 %)  14 (2 %) | 7 (32 %)  7 (32 %)  3 (14 %)  5 (23 %)  6 (27 %)  3 (14 %)  0 (0 %)  4 (18 %)  16 (73 %)  0 (0 %)  12 (55 %)  3 (14 %) | 1.00¶  1.00¶  1.00¶  0.36¶  0.24¶  0.41¶  1.00¶  0.77¶  0.27¶  1.00¶  **<0.001**¶  **0.009**¶ | 47 (30 %)  53 (34 %)  19 (12 %)  29 (18 %)  18 (11 %)  33 (21 %)  11 (7 %)  58 (37 %)  88 (56 %)  1 (1 %)  66 (42 %)  15 (10 %) | 1 (17 %)  1 (17 %)  0 (0 %)  1 (17 %)  0 (0 %)  0 (0 %)  1 (17 %)  3 (50 %)  6 (100 %)  0 (0 %)  6 (100 %)  2 (33 %) | 0.67¶  0.67¶  1.00¶  1.00¶  1.00¶  0.60¶  0.37¶  0.67¶  **0.04**¶  1.00¶  **0.006**¶  **0.12**¶ |

Bold values indicate significant differences. SSBE, short-segment Barrett’s esophagus;BMI, body mass index; BW, body weight; BP, blood pressure; TG, triglyceride; HDL-C, high-density lipoprotein cholesterol; LDL-C, low-density lipoprotein cholesterol; Hb-A1c, hemoglobin A1c; HOMA-R, homeostatic model assessment of insulin resistance; UA, uric acid; ¶, Fisher’s exact test: †, χ^2^ test: ‡, Student’s t-test.

***Table G. Factors associated with reflux esophagitis or Barrett’s esophagus in non-obese men (Visceral fat area <100 cm^2^)***

|  | Age-adjusted analysis¶ | | Multivariable analysis† | |
| --- | --- | --- | --- | --- |
|  | OR | (95% CI) | OR | (95% CI) |
| *Reflux esophagitis (presence vs. absence)*  BMI (per 5 kg/m^2^)  Visceral fat area (per 50 cm^2^)  TG (per 50 mg/dL)  Current smoking  No breakfast ≥ 3 times / week  Proton pump inhibitor use  Aspirin use  Sleeping < 6 hours/night  Hiatus hernia  SSBE | 1.30  **1.88**  1.15  **2.44**  1.37  1.83 ^▲^  0.33  1.10  **10.6**  **5.10** | (0.76 – 2.20)  (1.01 – 3.49)  (0.94 – 1.40)  (1.40 – 4.27)  (0.77 – 2.44)  (0.92 – 3.64)  (0.08 – 1.38)  (0.66 – 1.85)  (5.82 – 19.3)  (2.46 – 10.6) | **3.10**  **8.34**  **2.27** | (1.71 – 5.61)  (4.59 – 15.2)  (1.06 – 4.90) |
| *Reflux esophagitis (LA-A vs.B* – *D)*  BMI (per 5 kg/m^2^)  Waist circumference (per 5 cm)  LDL-C (per 20 mg/dL)  UA (per 1 mg/dL)  Alcohol consumption ≥ 40g /day  Alcohol drinking habit ≥5 days/week  Proton pump inhibitor use | 0.23  0.78  0.73  1.52 ^▲^  3.34 ^▲^  3.30 ^▲^  3.26 ^▲^ | (0.03 – 1.62)  (0.42 – 1.45)  (0.49 – 1.09)  (0.94 – 2.45)  (0.97 – 11.4)  (0.99 – 10.7)  (0.85 – 12.5) | **3.38** | (1.04 – 11.0) |
| *Barrett’s esophagus (presence vs absence)*  HDL-C (per 20 mg/dL)  Alcohol consumption ≥ 40g /day  Alcohol drinking habit ≥5 days/week  Proton pump inhibitor use  Statin use  Hiatus hernia  Reflux esophagitis | 1.34  **2.23**  **1.97**  2.10 ^▲^  1.80  **7.50**  **5.02** | (0.87 – 2.06)  (1.17 – 4.24)  (1.07 – 3.63)  (0.94 – 4.67)  (0.87 – 3.74)  (3.73 – 15.1)  (2.42 – 10.4) | **1.92**  **6.40**  **2.20** | (1.02 – 3.62)  (3.11 – 13.2)  (1.03 – 4.68) |

Bold values indicate significant differences. ^▲^, p < 0.1; OR, odds ratio; CI, confident interval; BMI, body mass index; TG, triglyceride; HDL-C, high-density lipoprotein cholesterol; LDL-C, low-density lipoprotein cholesterol; UA, uric acid; SSBE, short-segment Barrett’s esophagus; ¶, logistic regression analysis adjusted for age, †, logistic regression analysis adjusted for factors selected by stepwise method form marginally associated factors in age-adjusted analyses (p < 0.1)

***Table H. Factors associated with reflux esophagitis or Barrett’s esophagus in obese men (Visceral fat area ≥100 cm^2^)***

|  | Age-adjusted analysis¶ | | Multivariable analysis† | |
| --- | --- | --- | --- | --- |
|  | OR | (95% CI) | OR | (95% CI) |
| *Reflux esophagitis (presence vs. absence)*  BMI (per 5 kg/m^2^)  Waist circumference (per 5 cm)  ≥ 10kg of BW increase since 20 y/o  Diastolic BP (per 10 mmHg)  TG (per 50 mg/dL)  HOMA-R (per 1)  UA (per 1 mg/dL)  Alcohol consumption ≥ 40g /day  Late dinner ≥ 3 times / week  Statin use  Sleeping < 6 hours/night  Hiatus hernia  SSBE | 1.16  1.08  **1.76**  **1.33**  **1.10**  1.07  1.16 ^▲^  1.32  1.55 ^▲^  0.65 ^▲^  **1.59**  **8.39**  **2.32** | (0.84 – 1.62)  (0.95 – 1.23)  (1.06 – 2.93)  (1.08 – 1.64)  (1.00 – 1.19)  (0.97 – 1.18)  (0.98 – 1.37)  (0.86 – 2.02)  (0.97 – 2.46)  (0.39 – 1.08)  (1.04 – 2.44)  (5.08 – 13.9)  (1.28 – 4.20) | **1.15**  **1.79**  **9.32** | (1.03 – 1.27)  (1.15 – 2.80)  (5.58 – 15.6) |
| *Reflux esophagitis (LA-A vs.B* – *D)*  BMI (per 5 kg/m^2^)  Waist circumference (per 5 cm)  Visceral fat area (per 50 cm^2^)  Systolic BP (per 10 mmHg)  Diastolic BP (per 10 mmHg)  ≥ 1 hour walk / day  Statin use  Hiatus hernia  SSBE | **3.31**  **1.78**  **2.09**  **1.39**  **1.51**  0.35 ^▲^  0.30  **8.37**  **5.01** | (1.40 – 7.82)  (1.22 – 2.61)  (1.17 – 3.75)  (1.05 – 1.85)  (1.01 – 2.26)  (0.11 – 1.13)  (0.06 – 1.38)  (1.06 – 66.2)  (1.65 – 15.2) | **2.20**  6.71 ^▲^  **4.36** | (1.14 – 4.25)  (0.81 – 55.3)  (1.36 – 14.0) |
| *Barrett’s esophagus (presence vs absence)*  Systolic BP (per 10 mmHg)  Diastolic BP (per 10 mmHg)  LDL-C (per 20 mg/dL)  HOMA-R (per 1)  Alcohol consumption ≥ 40g /day  ≥ 1 hour walk / day  Aspirin use  Sleeping < 6 hours/night  Hiatus hernia  Reflux esophagitis | 1.17 ^▲^  1.22  1.17 ^▲^  1.08  1.59 ^▲^  0.62  0.35 ^▲^  1.47  **6.16**  **2.31** | (1.00 – 1.37)  (0.94 – 1.59)  (0.99 – 1.40)  (0.97 – 1.21)  (0.93 – 2.71)  (0.34 – 1.14)  (0.11 – 1.15)  (0.86 – 2.52)  (3.35 – 11.3)  (1.28 – 4.19) | **6.17** | (3.35 – 11.4) |

Bold values indicate significant differences. ^▲^, p < 0.1; OR, odds ratio; CI, confident interval; BMI, body mass index; BW, body weight; BP, blood pressure; TG, triglyceride; LDL-C, low-density lipoprotein cholesterol; HOMA-R, homeostatic model assessment of insulin resistance; UA, uric acid; SSBE, short-segment Barrett’s esophagus; ¶, logistic regression analysis adjusted for age, †, logistic regression analysis adjusted for factors selected by stepwise method form marginally associated factors in age-adjusted analyses (p < 0.1)

***Table I. Factors associated with reflux esophagitis or Barrett’s esophagus in non-obese women (Visceral fat area <100 cm^2^)***

|  | Age-adjusted analysis¶ | | Multivariable analysis† | |
| --- | --- | --- | --- | --- |
|  | OR | (95% CI) | OR | (95% CI) |
| *Reflux esophagitis (presence vs. absence)*  BMI (per 5 kg/m^2^)  Visceral fat area (per 50 cm^2^)  Systolic BP (per 10 mmHg)  TG (per 50 mg/dL)  LDL-C (per 20 mg/dL)  Hb-A1c (per 1 %)  ≥ 1 hour walk / day  No breakfast ≥ 3 times / week  Hiatus hernia  SSBE | 1.92  **3.82**  1.16  1.18  1.19  1.42  2.27 ^▲^  N. A.  **13.4**  **8.56** | (0.82 – 4.50)  (1.23 – 11.9)  (0.90 – 1.51)  (0.93 – 1.49)  (0.87 – 1.63)  (0.52 – 3.87)  (0.86 – 5.96)  (4.12 – 43.5)  (2.25 – 32.6) | **3.58**  **9.92**  **5.48** | (1.09 – 11.7)  (3.11 – 31.6)  (1.29 – 23.2) |
| *Reflux esophagitis (LA-A vs.B* – *D)*  ≥ 10kg of BW increase since 20 y/o  Hb-A1c (per 1 %)  UA (per 1 mg/dL)  Sweating for 30 mins twice a week  Statin use | 16.4  10.8  5.05  44.5 ^▲^  7.33 | (0.41 – 664)  (0.10 – 1174)  (0.36 – 71.0)  (0.84 – 2344)  (0.48 – 111) |  |  |
| *Barrett’s esophagus (presence vs absence)*  BMI (per 5 kg/m^2^)  LDL-C (per 20 mg/dL)  UA (per 1 mg/dL)  Hiatus hernia  Reflux esophagitis | 0.63  1.29 ^▲^  1.31  **5.30**  **8.50** | (0.26 – 1.53)  (0.98 – 1.69)  (0.88 – 1.95)  (2.15 – 13.1)  (2.24 – 32.4) | **4.25**  **4.17** | (1.73 – 10.4)  (1.03 – 16.9) |

Bold values indicate significant differences. ^▲^, p < 0.1; OR, odds ratio; CI, confident interval; BMI, body mass index; BW, body weight; BP, blood pressure; TG, triglyceride; LDL-C, low-density lipoprotein cholesterol; Hb-A1c, hemoglobin A1c; UA, uric acid; SSBE, short-segment Barrett’s esophagus; ¶, logistic regression analysis adjusted for age, †, logistic regression analysis adjusted for factors selected by stepwise method form marginally associated factors in age-adjusted analyses (p < 0.1)

***Table J. Factors associated with reflux esophagitis or Barrett’s esophagus in obese women (Visceral fat area ≥100 cm^2^)***

|  | Age-adjusted analysis¶ | | Multivariable analysis† | |
| --- | --- | --- | --- | --- |
|  | OR | (95% CI) | OR | (95% CI) |
| *Reflux esophagitis (presence vs. absence)*  Visceral fat area (per 50 cm^2^)  LDL-C (per 20 mg/dL)  Alcohol consumption ≥ 40g /day  ≥ 1 hour walk / day  Sleeping < 6 hours/night  Hiatus hernia  SSBE | 1.79  1.19  2.61  0.43  2.57  **8.57**  5.38 ^▲^ | (0.87 – 3.70)  (0.90 – 1.57)  (0.46 – 14.7)  (0.12 – 1.60)  (0.80 – 8.28)  (2.25 – 32.6)  (0.88 – 32.7) | **7.16** | (1.97 – 26.0) |
| *Reflux esophagitis (LA-A vs.B* – *D)*  Waist circumference (per 5 cm) | 0.13 | (0.01 – 1.91) |  |  |
| *Barrett’s esophagus (presence vs absence)*  Waist circumference (per 5 cm)  Sleeping < 6 hours/night  Hiatus hernia  Reflux esophagitis | 0.63  N. A.  N. A.  5.59 ^▲^ | (0.34 – 1.12)  (0.90 – 34.8) |  |  |

Bold values indicate significant differences. ^▲^, p < 0.1; OR, odds ratio; CI, confident interval; LDL-C, low-density lipoprotein cholesterol; SSBE, short-segment Barrett’s esophagus; ¶, logistic regression analysis adjusted for age, †, logistic regression analysis adjusted for factors selected by stepwise method form marginally associated factors in age-adjusted analyses (p < 0.1)
